# Iodido­bis­(morpholine-4-carbodi­thio­ato-κ^2^ *S*,*S*′)(1,10-phenanthroline-κ^2^ *N*,*N*′)bis­muth(III)

**DOI:** 10.1107/S1600536813033515

**Published:** 2013-12-14

**Authors:** Feng Li, Handong Yin, Guoxing Wu

**Affiliations:** a Department of Chemistry and Chemical Engineering, Lvliang University, Lishi Shanxi 033000, People’s Republic of China; b College of Chemistry and Chemical Engineering, Liaocheng University, Shandong 252059, People’s Republic of China

## Abstract

The title compound, [Bi(C_4_H_8_NOS_2_)_2_I(C_12_H_8_N_2_)], is monomeric, with the Bi^III^ atom chelated by the two S atoms of two morpholine-4-carbodi­thio­ate ligands and the two N atoms of a 1,10-phenanthroline ligand. An iodide ligand completes the coordination sphere, with the seven-coordinate Bi^III^ atom adopting a highly distorted monocapped octa­hedral geometry.

## Related literature   

For di­thio­carbamates as ligands to transition metals, see: Xu *et al.* (2001 ▶); Bardaji *et al.* (1994 ▶). For bis­muth(III)–di­thio­carbamate complexes, see: Yin *et al.* (2003 ▶). For related Bi/N structures, see: Baraanyi *et al.* (1977 ▶).
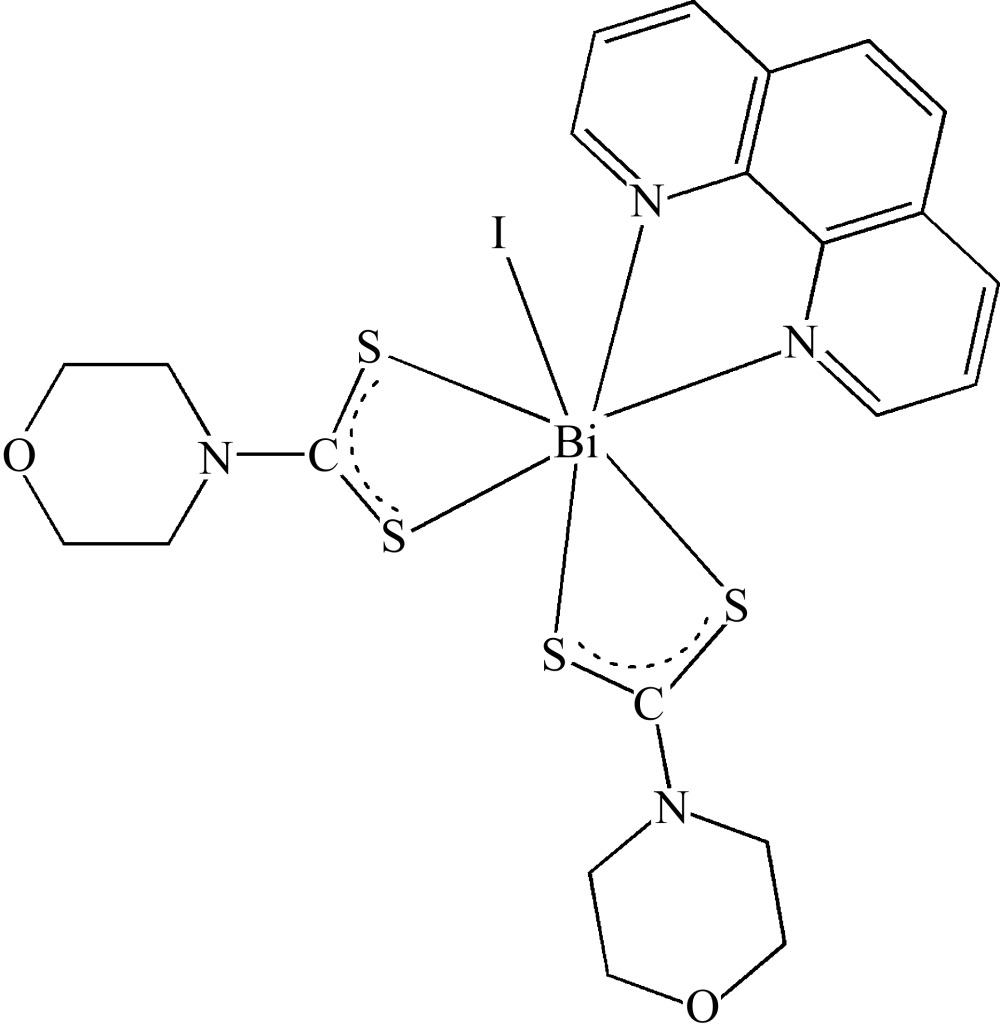


## Experimental   

### 

#### Crystal data  

[Bi(C_4_H_8_NOS_2_)_2_I(C_12_H_8_N_2_)]
*M* *_r_* = 836.62
Monoclinic, 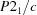

*a* = 14.782 (4) Å
*b* = 10.883 (3) Å
*c* = 18.030 (4) Å
β = 100.035 (4)°
*V* = 2856.2 (12) Å^3^
*Z* = 4
Mo *K*α radiation
μ = 7.57 mm^−1^
*T* = 298 K
0.52 × 0.42 × 0.13 mm

#### Data collection  

Siemens SMART CCD area-detector diffractometer
Absorption correction: multi-scan (*SADABS*; Sheldrick, 1996 ▶) *T*_min_ = 0.111, *T*_max_ = 0.440
14581 measured reflections
5013 independent reflections
3883 reflections with *I* > 2σ(*I*)
*R*_int_ = 0.051

#### Refinement  

*R*[*F* ^2^ > 2σ(*F* ^2^)] = 0.036
*wR*(*F* ^2^) = 0.100
*S* = 1.00
5013 reflections
307 parameters
162 restraints
H-atom parameters constrained
Δρ_max_ = 2.14 e Å^−3^
Δρ_min_ = −1.18 e Å^−3^

### 

Data collection: *SMART* (Siemens, 1996 ▶); cell refinement: *SAINT* (Siemens, 1996 ▶); data reduction: *SAINT*; program(s) used to solve structure: *SHELXS97* (Sheldrick, 2008 ▶); program(s) used to refine structure: *SHELXL97* (Sheldrick, 2008 ▶); molecular graphics: *SHELXTL* (Sheldrick, 2008 ▶); software used to prepare material for publication: *SHELXTL*.

## Supplementary Material

Crystal structure: contains datablock(s) I, New_Global_Publ_Block. DOI: 10.1107/S1600536813033515/sj5375sup1.cif

Structure factors: contains datablock(s) I. DOI: 10.1107/S1600536813033515/sj5375Isup2.hkl

Additional supporting information:  crystallographic information; 3D view; checkCIF report

## Figures and Tables

**Table 1:** Selected bond lengths (Å)

Bi1—S3	2.683 (2)
Bi1—S1	2.7032 (18)
Bi1—N3	2.738 (6)
Bi1—S2	2.775 (2)
Bi1—N4	2.831 (6)
Bi1—S4	2.962 (2)
Bi1—I1	3.1043 (9)

**Fig. 1.**
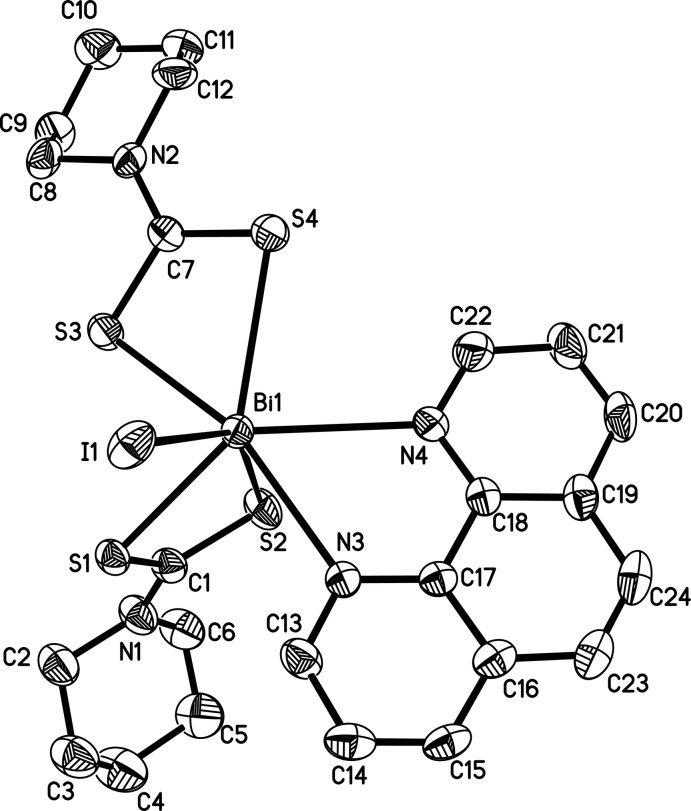
The structure of the title complex, showing 30% probability displacement ellipsoids and the atom-numbering scheme. H atoms have been omitted for clarity.

## supplementary crystallographic information

### 1. Comment

Dithiocarbamates have been known as effective ligands for transition metal ions for many years. They can form chelates (Xu *et al.*, 2001) or act as bridging ligands (Bardaji *et al.*, 1994). However, the chemistry of main-group metal complexes with dithiocarbamates has been less extensively studied, and only a few reports describing bismuth(III) dithiocarbamate complexes have appeared (Yin *et al.*, 2003). As a continuation of our interest in sulfur-containing ligands, we report here the synthesis and structure of the title compound, (I).

The title compound, (I), is monomeric, with the Bi atom chelated by the S atoms of two morpholine-4-carbodithioate ligands and the two N atoms of 1,10-phenanthroline. An iodido ligand completes the coordination environment of the seven coordinate Bi atom (Fig. 1). The Bi atom is in a capped octahedral environment, with atoms S3 and N3 in axial positions, and atoms S1, S2, S4 and I1 in the equatorial plane. The remaining N atom (N4) of the 1,10-phenanthroline ligand caps the S2/S4/N3 face of this octahedron, giving a highly distorted capped octahedral coordination geometry. One of the bidentate pyrrolidinyldithiocarbamate ligands forms a significantly longer Bi—S bond [Bi1—S4 = 2.962 (2) Å] than the others in the complex. This variation in coordination strength is also signalled by the fact that the C7—S4 bond is significantly shorter than the other C—S bonds, suggesting some delocalization in the system. In addition, the chelating phenanthroline ligand is bound to the Bi atom through both of its N atoms. The Bi1—N3 and Bi1—N4 distances fall in the same range as in other Bi/N complexes (Baraanyi *et al.*, 1977).

### 2. Experimental

To a stirred solution of BiI_3_ (0.15 mmol) in acetonitrile (*ca* 20 ml), phenanthroline (0.15 mmol) and sodium morpholine-4-carbodithioate (0.30 mmol) were added. A yellow coloured solution was obtained and, after concentration and cooling, small yellow crystals of the title compound were obtained. These were collected and dried in a vacuum [yield 82%, m.p. 452 K]. Analysis calculated for C_24_H_28_BiIN_4_S_4_: C 34.45, H 3.37, N 6.70%; found: C 32.78, H 3.24, N 6.77%.

### 3. Refinement

All H atoms were positioned geometrically and treated as riding on their parent atoms [C—H = 0.93 Å and *U*_iso_=1.2Ueq (C) for aromatic, C—H =0.97 Å and *U*_iso_ = 1.2Ueq (C) for CH_2_ H atoms].

### Crystal data

**Table:**

[Bi(C_4_H_8_NOS_2_)_2_I(C_12_H_8_N_2_)]	*F*(000) = 1600
*M**_r_* = 836.62	*D*_x_ = 1.946 Mg m^−^^3^
Monoclinic, *P*2_1_/*c*	Mo *K*α radiation, λ = 0.71073 Å
Hall symbol: -P 2ybc	Cell parameters from 4986 reflections
*a* = 14.782 (4) Å	θ = 2.2–25.8°
*b* = 10.883 (3) Å	µ = 7.57 mm^−^^1^
*c* = 18.030 (4) Å	*T* = 298 K
β = 100.035 (4)°	Block, orange-red
*V* = 2856.2 (12) Å^3^	0.52 × 0.42 × 0.13 mm
*Z* = 4	

### Data collection

**Table:**

Siemens SMART CCD area-detector diffractometer	5013 independent reflections
Radiation source: fine-focus sealed tube	3883 reflections with *I* > 2σ(*I*)
Graphite monochromator	*R*_int_ = 0.051
φ and ω scans	θ_max_ = 25.0°, θ_min_ = 2.2°
Absorption correction: multi-scan (*SADABS*; Sheldrick, 1996)	*h* = −17→17
*T*_min_ = 0.111, *T*_max_ = 0.440	*k* = −12→12
14581 measured reflections	*l* = −19→21

### Refinement

**Table:**

Refinement on *F*^2^	Primary atom site location: structure-invariant direct methods
Least-squares matrix: full	Secondary atom site location: difference Fourier map
*R*[*F*^2^ > 2σ(*F*^2^)] = 0.036	Hydrogen site location: inferred from neighbouring sites
*wR*(*F*^2^) = 0.100	H-atom parameters constrained
*S* = 1.00	*w* = 1/[σ^2^(*F*_o_^2^) + (0.0573*P*)^2^ + 0.7329*P*] where *P* = (*F*_o_^2^ + 2*F*_c_^2^)/3
5013 reflections	(Δ/σ)_max_ = 0.001
307 parameters	Δρ_max_ = 2.14 e Å^−^^3^
162 restraints	Δρ_min_ = −1.18 e Å^−^^3^

### Special details

**Table:**

Geometry. All e.s.d.'s (except the e.s.d. in the dihedral angle between two l.s. planes) are estimated using the full covariance matrix. The cell e.s.d.'s are taken into account individually in the estimation of e.s.d.'s in distances, angles and torsion angles; correlations between e.s.d.'s in cell parameters are only used when they are defined by crystal symmetry. An approximate (isotropic) treatment of cell e.s.d.'s is used for estimating e.s.d.'s involving l.s. planes.
Refinement. Refinement of *F*^2^ against ALL reflections. The weighted *R*-factor *wR* and goodness of fit *S* are based on *F*^2^, conventional *R*-factors *R* are based on *F*, with *F* set to zero for negative *F*^2^. The threshold expression of *F*^2^ > σ(*F*^2^) is used only for calculating *R*-factors(gt) *etc*. and is not relevant to the choice of reflections for refinement. *R*-factors based on *F*^2^ are statistically about twice as large as those based on *F*, and *R*- factors based on ALL data will be even larger.

### Fractional atomic coordinates and isotropic or equivalent isotropic displacement parameters (Å^2^)

**Table:**

	*x*	*y*	*z*	*U*_iso_*/*U*_eq_	
Bi1	0.239399 (17)	0.06921 (2)	0.229937 (14)	0.04234 (11)	
I1	0.14675 (4)	0.32613 (5)	0.21768 (3)	0.06751 (18)	
N1	0.5244 (5)	0.0651 (5)	0.1730 (4)	0.0645 (17)	
N2	0.2870 (4)	0.0260 (6)	0.4879 (3)	0.0530 (15)	
N3	0.1809 (4)	0.0545 (5)	0.0775 (3)	0.0480 (14)	
N4	0.1352 (4)	−0.1340 (5)	0.1647 (3)	0.0527 (15)	
S1	0.38192 (12)	0.19983 (16)	0.19532 (10)	0.0474 (4)	
S2	0.38367 (15)	−0.07187 (17)	0.19689 (14)	0.0632 (6)	
S3	0.33747 (13)	0.1194 (2)	0.36628 (11)	0.0568 (5)	
S4	0.17373 (15)	−0.0365 (2)	0.36236 (12)	0.0680 (6)	
C1	0.4392 (5)	0.0635 (6)	0.1872 (4)	0.0510 (17)	
C2	0.5748 (6)	0.1789 (8)	0.1643 (6)	0.075 (2)	
H2A	0.5378	0.2489	0.1736	0.090*	
H2B	0.6310	0.1805	0.2011	0.090*	
C3	0.5971 (7)	0.1885 (8)	0.0886 (6)	0.088 (2)	
H3A	0.6322	0.2628	0.0846	0.106*	
H3B	0.5409	0.1929	0.0517	0.106*	
C4	0.6531 (9)	0.0761 (9)	0.0728 (8)	0.105 (3)	
H4A	0.7120	0.0763	0.1065	0.126*	
H4B	0.6644	0.0792	0.0215	0.126*	
C5	0.5999 (8)	−0.0423 (9)	0.0847 (6)	0.091 (3)	
H5A	0.5443	−0.0463	0.0471	0.110*	
H5B	0.6373	−0.1132	0.0780	0.110*	
C6	0.5759 (7)	−0.0457 (8)	0.1590 (6)	0.078 (2)	
H6A	0.6314	−0.0515	0.1965	0.093*	
H6B	0.5389	−0.1180	0.1636	0.093*	
C7	0.2667 (5)	0.0334 (6)	0.4124 (4)	0.0460 (16)	
C8	0.3662 (6)	0.0842 (7)	0.5339 (4)	0.060 (2)	
H8A	0.3453	0.1427	0.5678	0.072*	
H8B	0.4007	0.1289	0.5015	0.072*	
C9	0.4270 (6)	−0.0084 (8)	0.5785 (5)	0.066 (2)	
H9A	0.4773	0.0334	0.6103	0.079*	
H9B	0.4529	−0.0617	0.5445	0.079*	
C10	0.3754 (7)	−0.0844 (8)	0.6265 (5)	0.078 (3)	
H10A	0.3571	−0.0330	0.6653	0.093*	
H10B	0.4152	−0.1487	0.6511	0.093*	
C11	0.2915 (7)	−0.1413 (8)	0.5798 (5)	0.076 (3)	
H11A	0.2559	−0.1826	0.6128	0.091*	
H11B	0.3104	−0.2024	0.5465	0.091*	
C12	0.2312 (6)	−0.0449 (9)	0.5330 (4)	0.071 (3)	
H12A	0.1811	−0.0852	0.4999	0.085*	
H12B	0.2050	0.0099	0.5660	0.085*	
C13	0.1992 (5)	0.1464 (8)	0.0334 (5)	0.060 (2)	
H13	0.2324	0.2130	0.0561	0.072*	
C14	0.1719 (6)	0.1489 (9)	−0.0440 (5)	0.066 (2)	
H14	0.1868	0.2154	−0.0719	0.079*	
C15	0.1235 (5)	0.0534 (8)	−0.0783 (4)	0.059 (2)	
H15	0.1050	0.0537	−0.1303	0.070*	
C16	0.1010 (5)	−0.0468 (7)	−0.0353 (4)	0.0534 (19)	
C17	0.1315 (4)	−0.0417 (6)	0.0438 (4)	0.0435 (16)	
C18	0.1079 (4)	−0.1412 (6)	0.0895 (4)	0.0448 (16)	
C19	0.0559 (5)	−0.2401 (7)	0.0535 (5)	0.056 (2)	
C20	0.0328 (6)	−0.3350 (7)	0.1006 (6)	0.069 (2)	
H20	−0.0014	−0.4021	0.0796	0.083*	
C21	0.0604 (6)	−0.3282 (7)	0.1759 (6)	0.074 (3)	
H21	0.0450	−0.3900	0.2071	0.088*	
C22	0.1124 (6)	−0.2273 (8)	0.2064 (5)	0.072 (2)	
H22	0.1323	−0.2248	0.2582	0.087*	
C23	0.0497 (5)	−0.1488 (9)	−0.0675 (5)	0.066 (2)	
H23	0.0304	−0.1511	−0.1194	0.080*	
C24	0.0282 (6)	−0.2412 (8)	−0.0262 (5)	0.069 (2)	
H24	−0.0052	−0.3074	−0.0494	0.083*	

### Atomic displacement parameters (Å^2^)

**Table:**

	*U*^11^	*U*^22^	*U*^33^	*U*^12^	*U*^13^	*U*^23^
Bi1	0.04840 (17)	0.03802 (17)	0.03946 (17)	−0.00219 (11)	0.00451 (12)	0.00165 (11)
I1	0.0847 (4)	0.0605 (4)	0.0538 (3)	0.0195 (3)	0.0024 (3)	−0.0045 (2)
N1	0.068 (4)	0.046 (3)	0.086 (4)	0.001 (3)	0.031 (3)	0.001 (3)
N2	0.065 (4)	0.056 (4)	0.036 (3)	−0.010 (3)	0.002 (3)	−0.003 (3)
N3	0.051 (3)	0.048 (4)	0.045 (3)	−0.006 (3)	0.006 (3)	0.001 (3)
N4	0.066 (4)	0.045 (4)	0.050 (4)	−0.011 (3)	0.016 (3)	0.001 (3)
S1	0.0550 (10)	0.0340 (9)	0.0541 (11)	−0.0003 (8)	0.0121 (8)	0.0044 (8)
S2	0.0688 (13)	0.0349 (10)	0.0890 (16)	0.0004 (9)	0.0228 (12)	0.0065 (9)
S3	0.0581 (11)	0.0625 (12)	0.0457 (11)	−0.0173 (10)	−0.0026 (9)	0.0103 (9)
S4	0.0658 (13)	0.0913 (16)	0.0444 (11)	−0.0299 (12)	0.0026 (10)	0.0025 (11)
C1	0.061 (4)	0.039 (4)	0.055 (4)	0.003 (3)	0.013 (3)	0.007 (3)
C2	0.080 (5)	0.057 (4)	0.094 (5)	−0.004 (4)	0.034 (4)	0.000 (4)
C3	0.108 (6)	0.065 (5)	0.100 (6)	0.010 (5)	0.040 (5)	0.014 (4)
C4	0.122 (7)	0.084 (6)	0.124 (7)	0.021 (5)	0.063 (6)	0.012 (5)
C5	0.112 (6)	0.065 (5)	0.103 (6)	0.023 (5)	0.033 (5)	0.004 (5)
C6	0.084 (5)	0.059 (4)	0.098 (5)	0.014 (4)	0.038 (4)	0.008 (4)
C7	0.043 (4)	0.044 (4)	0.051 (4)	0.003 (3)	0.008 (3)	0.007 (3)
C8	0.074 (5)	0.059 (5)	0.040 (4)	−0.004 (4)	−0.010 (4)	−0.001 (4)
C9	0.061 (5)	0.070 (6)	0.060 (5)	0.001 (4)	−0.007 (4)	0.010 (4)
C10	0.087 (7)	0.083 (7)	0.061 (6)	0.003 (5)	0.005 (5)	0.025 (5)
C11	0.099 (7)	0.072 (6)	0.060 (6)	−0.020 (5)	0.021 (5)	0.012 (5)
C12	0.069 (5)	0.106 (7)	0.039 (4)	−0.018 (5)	0.012 (4)	0.009 (4)
C13	0.057 (5)	0.060 (5)	0.061 (5)	−0.008 (4)	0.003 (4)	0.017 (4)
C14	0.063 (5)	0.081 (6)	0.055 (5)	0.007 (4)	0.014 (4)	0.021 (5)
C15	0.056 (5)	0.082 (6)	0.036 (4)	0.014 (4)	0.005 (4)	0.006 (4)
C16	0.041 (4)	0.074 (5)	0.046 (4)	0.012 (4)	0.008 (3)	−0.003 (4)
C17	0.037 (3)	0.050 (4)	0.045 (4)	0.007 (3)	0.012 (3)	−0.001 (3)
C18	0.038 (4)	0.042 (4)	0.055 (5)	0.003 (3)	0.010 (3)	−0.004 (3)
C19	0.045 (4)	0.049 (5)	0.076 (6)	0.001 (3)	0.012 (4)	−0.018 (4)
C20	0.070 (5)	0.043 (5)	0.094 (7)	−0.016 (4)	0.015 (5)	−0.024 (5)
C21	0.090 (6)	0.039 (5)	0.097 (8)	−0.014 (4)	0.029 (6)	−0.007 (5)
C22	0.098 (7)	0.059 (5)	0.063 (6)	−0.010 (5)	0.024 (5)	−0.007 (4)
C23	0.057 (5)	0.075 (6)	0.063 (5)	0.003 (4)	−0.001 (4)	−0.023 (5)
C24	0.057 (5)	0.062 (6)	0.083 (7)	−0.003 (4)	−0.004 (4)	−0.028 (5)

### Geometric parameters (Å, º)

**Table:**

Bi1—S3	2.683 (2)	C8—C9	1.489 (11)
Bi1—S1	2.7032 (18)	C8—H8A	0.9700
Bi1—N3	2.738 (6)	C8—H8B	0.9700
Bi1—S2	2.775 (2)	C9—C10	1.499 (11)
Bi1—N4	2.831 (6)	C9—H9A	0.9700
Bi1—S4	2.962 (2)	C9—H9B	0.9700
Bi1—I1	3.1043 (9)	C10—C11	1.506 (13)
N1—C1	1.328 (9)	C10—H10A	0.9700
N1—C2	1.468 (10)	C10—H10B	0.9700
N1—C6	1.472 (10)	C11—C12	1.532 (12)
N2—C7	1.344 (9)	C11—H11A	0.9700
N2—C8	1.456 (10)	C11—H11B	0.9700
N2—C12	1.472 (9)	C12—H12A	0.9700
N3—C13	1.335 (9)	C12—H12B	0.9700
N3—C17	1.358 (9)	C13—C14	1.383 (11)
N4—C22	1.341 (10)	C13—H13	0.9300
N4—C18	1.347 (9)	C14—C15	1.349 (11)
S1—C1	1.727 (7)	C14—H14	0.9300
S2—C1	1.710 (7)	C15—C16	1.411 (11)
S3—C7	1.722 (7)	C15—H15	0.9300
S4—C7	1.689 (7)	C16—C23	1.411 (11)
C2—C3	1.463 (13)	C16—C17	1.420 (10)
C2—H2A	0.9700	C17—C18	1.440 (9)
C2—H2B	0.9700	C18—C19	1.413 (10)
C3—C4	1.531 (12)	C19—C20	1.416 (11)
C3—H3A	0.9700	C19—C24	1.424 (11)
C3—H3B	0.9700	C20—C21	1.350 (13)
C4—C5	1.544 (13)	C20—H20	0.9300
C4—H4A	0.9700	C21—C22	1.397 (11)
C4—H4B	0.9700	C21—H21	0.9300
C5—C6	1.445 (13)	C22—H22	0.9300
C5—H5A	0.9700	C23—C24	1.323 (12)
C5—H5B	0.9700	C23—H23	0.9300
C6—H6A	0.9700	C24—H24	0.9300
C6—H6B	0.9700		
			
S3—Bi1—S1	77.65 (6)	N2—C7—S4	122.1 (5)
S3—Bi1—N3	163.15 (13)	N2—C7—S3	118.4 (5)
S1—Bi1—N3	85.50 (13)	S4—C7—S3	119.5 (4)
S3—Bi1—S2	89.80 (7)	N2—C8—C9	111.3 (7)
S1—Bi1—S2	65.31 (6)	N2—C8—H8A	109.4
N3—Bi1—S2	82.66 (13)	C9—C8—H8A	109.4
S3—Bi1—N4	135.03 (13)	N2—C8—H8B	109.4
S1—Bi1—N4	134.78 (12)	C9—C8—H8B	109.4
N3—Bi1—N4	58.91 (17)	H8A—C8—H8B	108.0
S2—Bi1—N4	82.04 (13)	C8—C9—C10	111.4 (7)
S3—Bi1—S4	62.69 (6)	C8—C9—H9A	109.3
S1—Bi1—S4	140.22 (6)	C10—C9—H9A	109.3
N3—Bi1—S4	134.08 (12)	C8—C9—H9B	109.3
S2—Bi1—S4	109.28 (7)	C10—C9—H9B	109.3
N4—Bi1—S4	78.49 (12)	H9A—C9—H9B	108.0
S3—Bi1—I1	92.47 (5)	C9—C10—C11	110.9 (7)
S1—Bi1—I1	82.06 (4)	C9—C10—H10A	109.5
N3—Bi1—I1	85.58 (12)	C11—C10—H10A	109.5
S2—Bi1—I1	145.99 (4)	C9—C10—H10B	109.5
N4—Bi1—I1	118.20 (13)	C11—C10—H10B	109.5
S4—Bi1—I1	101.82 (5)	H10A—C10—H10B	108.1
C1—N1—C2	123.2 (6)	C10—C11—C12	111.7 (7)
C1—N1—C6	124.0 (6)	C10—C11—H11A	109.3
C2—N1—C6	112.7 (7)	C12—C11—H11A	109.3
C7—N2—C8	124.4 (6)	C10—C11—H11B	109.3
C7—N2—C12	122.9 (6)	C12—C11—H11B	109.3
C8—N2—C12	112.7 (6)	H11A—C11—H11B	107.9
C13—N3—C17	117.4 (6)	N2—C12—C11	109.6 (7)
C13—N3—Bi1	119.6 (5)	N2—C12—H12A	109.8
C17—N3—Bi1	123.1 (4)	C11—C12—H12A	109.8
C22—N4—C18	117.3 (7)	N2—C12—H12B	109.8
C22—N4—Bi1	122.0 (5)	C11—C12—H12B	109.8
C18—N4—Bi1	120.6 (4)	H12A—C12—H12B	108.2
C1—S1—Bi1	88.9 (2)	N3—C13—C14	124.3 (8)
C1—S2—Bi1	86.9 (3)	N3—C13—H13	117.9
C7—S3—Bi1	93.2 (3)	C14—C13—H13	117.9
C7—S4—Bi1	84.6 (2)	C15—C14—C13	119.0 (8)
N1—C1—S2	121.3 (5)	C15—C14—H14	120.5
N1—C1—S1	120.0 (5)	C13—C14—H14	120.5
S2—C1—S1	118.7 (4)	C14—C15—C16	120.1 (7)
C3—C2—N1	111.2 (8)	C14—C15—H15	120.0
C3—C2—H2A	109.4	C16—C15—H15	120.0
N1—C2—H2A	109.4	C23—C16—C15	122.9 (8)
C3—C2—H2B	109.4	C23—C16—C17	119.9 (8)
N1—C2—H2B	109.4	C15—C16—C17	117.2 (7)
H2A—C2—H2B	108.0	N3—C17—C16	122.1 (7)
C2—C3—C4	109.5 (8)	N3—C17—C18	119.2 (6)
C2—C3—H3A	109.8	C16—C17—C18	118.7 (7)
C4—C3—H3A	109.8	N4—C18—C19	123.4 (7)
C2—C3—H3B	109.8	N4—C18—C17	118.2 (6)
C4—C3—H3B	109.8	C19—C18—C17	118.4 (7)
H3A—C3—H3B	108.2	C18—C19—C20	116.6 (8)
C3—C4—C5	109.6 (8)	C18—C19—C24	120.6 (8)
C3—C4—H4A	109.7	C20—C19—C24	122.8 (8)
C5—C4—H4A	109.7	C21—C20—C19	120.1 (7)
C3—C4—H4B	109.7	C21—C20—H20	120.0
C5—C4—H4B	109.7	C19—C20—H20	120.0
H4A—C4—H4B	108.2	C20—C21—C22	119.2 (8)
C6—C5—C4	111.3 (9)	C20—C21—H21	120.4
C6—C5—H5A	109.4	C22—C21—H21	120.4
C4—C5—H5A	109.4	N4—C22—C21	123.3 (9)
C6—C5—H5B	109.4	N4—C22—H22	118.3
C4—C5—H5B	109.4	C21—C22—H22	118.3
H5A—C5—H5B	108.0	C24—C23—C16	122.1 (8)
C5—C6—N1	110.8 (8)	C24—C23—H23	119.0
C5—C6—H6A	109.5	C16—C23—H23	119.0
N1—C6—H6A	109.5	C23—C24—C19	120.4 (8)
C5—C6—H6B	109.5	C23—C24—H24	119.8
N1—C6—H6B	109.5	C19—C24—H24	119.8
H6A—C6—H6B	108.1		

## References

[bb1] Baraanyi, A. D., Cook, J. & Onyszchuk, M. (1977). *Inorg. Nucl. Chem. Lett.* **13**, 385–394.

[bb2] Bardaji, M., Connelly, N. G., Gimeno, M. C., Jimenez, J., Jones, P. G., Laguna, A. & Laguna, M. (1994). *J. Chem. Soc. Dalton Trans.* pp. 1163–1168.

[bb3] Sheldrick, G. M. (1996). *SADABS* University of Göttingen, Germany.

[bb4] Sheldrick, G. M. (2008). *Acta Cryst.* A**64**, 112–122. 10.1107/S0108767307043930 18156677

[bb5] Siemens (1996). *SMART* and *SAINT* Siemens Analytical X-ray Instruments Inc., Madison, Wisconsin, USA.

[bb6] Xu, L. Z., Zhao, P. S. & Zhang, S. S. (2001). *Chin. J. Chem.* **19**, 436–440.

[bb7] Yin, H. D., Wang, C. H. & Xing, Q. J. (2003). *Chin. J. Inorg. Chem.* **19**, 955–958.

